# Cross-Sectional Study for Detection and Risk Factor Analysis of ESBL-Producing Avian Pathogenic *Escherichia coli* Associated with Backyard Chickens in Pakistan

**DOI:** 10.3390/antibiotics12050934

**Published:** 2023-05-20

**Authors:** Muhammad Adnan Saeed, Muhammad Saqlain, Usman Waheed, Syed Ehtisham-ul-Haque, Aman Ullah Khan, Aziz ur Rehman, Muhammad Sajid, Farhan Ahmad Atif, Heinrich Neubauer, Hosny El-Adawy

**Affiliations:** 1 Department of Pathobiology, University of Veterinary and Animal Sciences, Lahore, CVAS Campus, 12-Km Chiniot Road, Jhang 35200, Pakistan; adnan.saeed@uvas.edu.pk (M.A.S.); saqlainzahoor514@gmail.com (M.S.); usman.waheed@uvas.edu.pk (U.W.); ehtishamsyed@uvas.edu.pk (S.E.-u.-H.); amanullah.khan@uvas.edu.pk (A.U.K.); aziz.rehman@uvas.edu.pk (A.u.R.); muhammad.sajid@uvas.edu.pk (M.S.); 2 Department of Clinical Sciences, University of Veterinary and Animal Sciences, Lahore, CVAS Campus, 12-Km Chiniot Road, Jhang 35200, Pakistan; farhan.atif@uvas.edu.pk; 3 Institute of Bacterial Infections and Zoonoses, Friedrich-Loeffler-Institut, 07743 Jena, Germany; heinrich.neubauer@fli.de; 4 Faculty of Veterinary Medicine, Kafrelsheikh University, Kafr El-Sheikh 35516, Egypt

**Keywords:** backyard chickens, extended-spectrum β-lactamase, *Escherichia coli*, risk factors, Pakistan

## Abstract

The increasing incidence of extended-spectrum β-lactamase (ESBL)-producing *Escherichia* (*E*.) *coli* in backyard chicken farming in Pakistan is of serious concern. This study aimed to assess the prevalence, antimicrobial resistance patterns and risk factors associated with ESBL avian pathogenic *E. coli* (APEC) isolated from backyard chickens in the Jhang district, Punjab, Pakistan. In total, 320 cloacal swabs were collected from four breeds of backyard chicken (Aseel, Golden, Misri and Necked Neck). ESBL *E. coli* were phenotypically identified using double disc synergy test (DDST) and corresponding genes were confirmed by multiplex polymerase chain reaction (mPCR). Out of the 320 samples, 164 (51.3%) were confirmed as *E. coli,* while 74 (45.1%) were characterized as ESBL *E. coli*. The frequency of isolation of ESBL *E. coli* was highest in Aseel chickens (35.1%). Of the 164 confirmed *E. coli*, 95.1%, 78.6%, 76.8%, 71.3%, 70.1%, 68.9%, 60.4% and 57.3% were resistant against tylosin, doxycycline, cefotaxime, enrofloxacin, colistin, trimethoprim/sulfamethoxazole, chloramphenicol and gentamicin, respectively. The ESBL gene types detected and their corresponding proportions were *bla*_CTX-M_ (54.1 %, 40/74), *bla*_TEM_, (12.2%, 9/74) and co-existence (*bla*_CTX-M_ and *bla*_TEM_) were shown in 33.8% (25/74). The *bla*_CTX-M_ gene sequence showed homology to *bla*_CTX-M-15_ from clinical isolates. The mean multiple antibiotic resistance index (MARI) was found to be higher among ESBL *E. coli* (0.25) when compared to non-ESBL *E. coli* (0.17). Both free-range husbandry management system (*p* = 0.02, OR: 30.00, 95% CI = 1.47–611.79) and high antimicrobial usage in the last 6 months (*p* = 0.01, OR: 25.17, 95% CI = 1.81–348.71) were found significantly associated with isolation of ESBL-producing *E. coli* in the tested samples using binary logistic regression analysis. This study confirmed the potential of backyard chickens as a reservoir for ESBL *E. coli* in the Jhang district, Punjab, Pakistan.

## 1. Introduction

Pathogenic and antibiotic-resistant bacteria are evolving rapidly in response to the intense global use of antimicrobial substances in clinical settings, animal production, veterinary medicine and the food industry [1]. Antimicrobial resistance (AMR) has a deep and unfavorable impact on the number of hospitalizations, mortality, burden for health care systems and economic losses [2]. *Escherichia coli* is a Gram-negative coccobacillus found as commensal in the gastrointestinal tract of many mammals, including humans and various species of animals and birds. Pathogenic strains of *E. coli* cause neonatal meningitis, septicemia, urinary tract infection (UTI) and severe gastroenteritis in humans [3]. Avian pathogenic *Escherichia coli* (APEC) causes colibacillosis, bronchitis, air sacculitis and swollen head syndrome in birds [4]. The emergence of extended-spectrum β-lactamase (ESBL) producing strains of *E. coli* jeopardize human and animal health and pose a risk to food safety and food security [5]. β-lactamases cause structural hydrolysis of the β-lactam ring of certain antibiotics via cleavage of amide bonds, e.g., the three major class A ESBL enzymes, including CTX-M, SHV and TEM [6,7]. Phenotypic resistance is widespread in otherwise susceptible *E. coli* strains due to the quick horizontal transfer of mobile genetic elements, including gene cassettes, plasmids and transposons [8].

ESBL-producing *E. coli* are transmitted to humans via the food chain or direct contact with the poultry reservoir [9]. Many countries still allow the use of antibacterial drugs for prophylaxis, improved feed efficiency and growth promotion [10,11]. In addition, non-judicious and irrational usage of antibacterial drugs inhibits vulnerable bacterial species resulting in the further spread of the resistant strains at the farm level [12].

People and their families dependent on backyard farming are in direct contact with chickens, which puts vulnerable individuals (children aged < 5 years and older people, 65 plus years of age) at risk of contracting pathogens [13]. It also favors the intraspecies (chicken-to-chicken) and trans-species spread of multidrug-resistant bacteria (MDR), especially in mixed farming systems [14]. ESBL-producing *E. coli* and *Salmonella*, and carbapenem-resistant strains of Gram-negative bacteria species, e.g., *Pseudomonas*, *Achromobacter* and *Acinetobacter*, are commonly isolated from backyard poultry and the farm environment, even from farms with no history of antibiotics usage [15].

In Pakistan, backyard poultry rearing is an important financial source for smallholder and rural farmers. Backyard poultry production is also a crucial segment for food security and economic vitality, along with the commercial poultry production system [16]. Since the share of Pakistani backyard, chicken farming in 2021–2022 (5.37%, 92.62 million units) is low in comparison to commercial chicken farming (94.63%, 1632.06 million units), the Government of Pakistan (GOP) is optimistic about enhancing the backyard chicken farming by supplying five million birds at subsidized rates to interested citizens via the “Prime Minister’s Initiative for Backyard Poultry Project,” which started in 2019 [17]. Data on the prevalence of ESBL *E. coli* in backyard chickens are scarce in Pakistan. The present study was designed to determine the prevalence of ESBL-producing *E. coli*, the molecular basis of AMR, the determination of susceptibility patterns and the analysis of risk-associated factors for backyard chicken farming in the Jhang district of the Punjab province, Pakistan.

## 2. Results

### 2.1. Isolation, Identification and Phenotypic Confirmation of ESBL Producing E. coli

Out of the 320 cloacal swabs collected from different breeds of backyard poultry in four tehsils of the Jhang district, 164 (51.3%) isolates were confirmed as *E. coli*. The overall prevalence of ESBL-producing *E. coli* was found as 23.1% (74/320) of samples from backyard poultry. The highest prevalence of 29.7% (22/74) ESBL *E. coli* was recorded in the tehsil of Jhang followed by 24.3% (18/74) in both the Shorkot and Athara Hazari tehsils, while 21.6% (16/74) prevalence was recorded in Ahmad Pur Sial. Regarding backyard chicken breeds, Aseel chickens showed the highest colonization of ESBL *E. coli* with a recovery rate of 35.1% (26/74), followed by 23% (17/74) in Golden chickens and Naked Neck chickens. The lowest prevalence of ESBL *E. coli* was found to be 19% (14/74) in Misri chickens.

### 2.2. Antimicrobial Susceptibility and Multiple Antibiotic Resistance Index (MARI) Profiling of E. coli

The phenotypic antimicrobial resistance of confirmed *E. coli* against 13 antimicrobial agents is summarized in Table 1. Out of 164 *E. coli*, 156 (95.1%) were resistant to tylosin. Higher resistance, i.e., ≥50%, were observed for doxycycline (78.6%), cefotaxime (76.8%), enrofloxacin (71.3%), colistin (70.1%), trimethoprim/sulfamethoxazole (68.9%), chloramphenicol (60.4%) and gentamicin (57.3%) (Table 1). The isolates were highly susceptible to imipenem, ciprofloxacin and neomycin, with 84.1%, 84% and 83.5%, respectively.

The geometric mean MARI of ESBL *E. coli* (0.25) was higher compared to the non-ESBL *E. coli* (0.17). The mean difference of MARI (0.08) revealed that the ESBL *E. coli* were resistant to one additional antibiotic compound as compared to non-ESBL *E. coli* on an average basis. The highest value of MARI was found to be 0.84 and 0.46 for ESBL *E. coli* and non-ESBL *E. coli*, respectively (Table 2).

### 2.3. Detection of ESBL Genes Using PCR and Sequence Analysis of bla_CTX-M_

**Figure 1:**
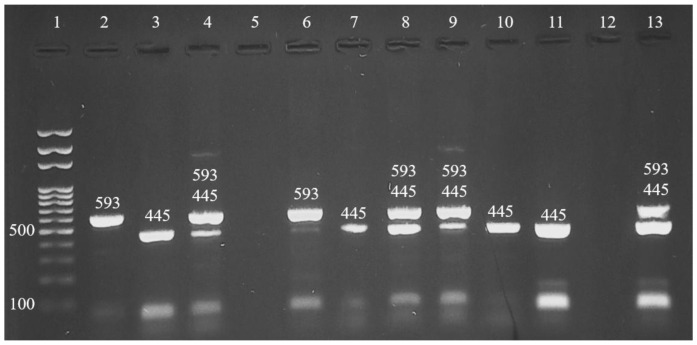
Agarose gel electrophoresis of mPCR profiles of *bla*_CTX-M_, *bla*_SHV_ and *bla*_TEM_ genes amplification of ESBL isolates: Well 1 (DNA ladder 100 bp, Solis BioDyne, Estonia); Wells 2 and 6 (*bla*_CTX-M_ gene, 593 bp); Wells 3, 7, 10, 11 (*bla*_TEM_ gene, 445 bp). Wells 4, 8, 9 and 13 (*bla*_CTX-M_ gene and *bla*_TEM_ gene). Wells 5 and 12 are negative samples.

Out of the 74 ESBL confirmed isolates, 40 (54.1%), 9 (12.2%) and 25 (33.8%) harbored *bla*_CTX-M_, *bla*_TEM_ and the co-existence of *bla*_CTX-M_ and *bla*_TEM_ genes, respectively (Figure 1). The *bla*_SHV_ gene was not identified in all investigated isolates by PCR. The highest detection frequency of *bla*_CTX-M_, *bla*_TEM_ and co-existence of *bla*_CTX-M_ and *bla*_TEM_ genes were recorded in Aseel chickens 36.5% (27/74), Golden chickens 5.4% (4/74) and Misri chickens 16.2% (12/74), respectively.

**Figure 2:**
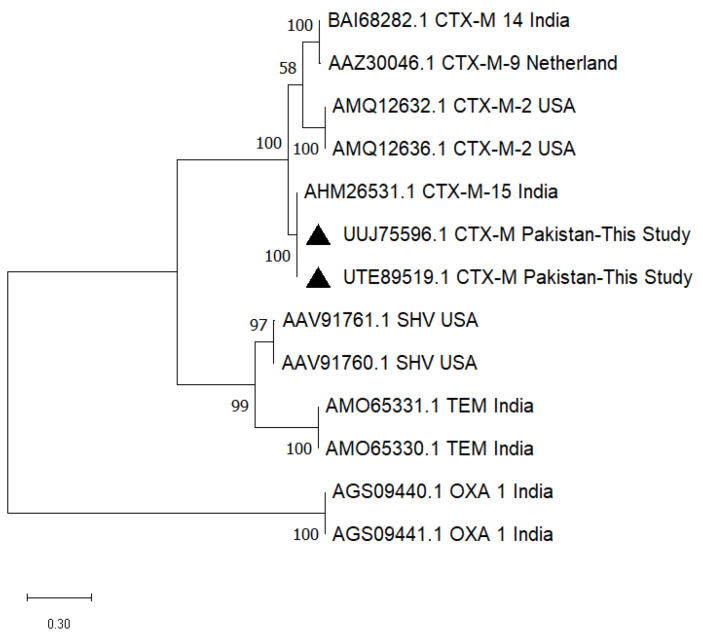
The phylogenetic tree constructed for β-lactamase sequences obtained in the present study (UTE89519.1, UUJ75596.1) was found to be similar to a CTX-M-15 type sequence from India. Black triangle represents the sequences obtained in this study.

Due to DNA quality and concentration and as nanodrop spectrophotometry (260/280 ratio) was not available, only two amplified PCR products of the *bla*_CTX-M_ gene could be sequenced, and the nucleotide sequence data were submitted to National Center for Biotechnology Information (NCBI) under accession numbers ON706023.1 and ON736876.1. A phylogenetic tree constructed with software MEGA 11 (64-bit) proved that the sequences (UTE89519.1, UUJ75596.1) obtained in the present study are close to the CTX-M-15 type (AHM26531.1) isolated from India (Eastern neighbor country of Pakistan) (Figure 2).

### 2.4. Risk Factors for the Presence of ESBL-Producing E. coli in Backyard Chicken, Jhang, Pakistan

The result of the prevalence of ESBL *E. coli* associated with the potential risk factors, e.g., location, chicken breed, sex, age, size of farm/unit birds, housing system, feeding resource, disinfection of drinking water, vaccination in last 6 months, contact with animals/birds, antimicrobial resistance level and antibacterial usage in last 6 months are presented in Table 3.

Out of 164 *E. coli* isolated in this study, 74 (45.1%) and 90 (54.9%) were identified as ESBL *E. coli* and Non-ESBL *E. coli*, respectively. High prevalence of ESBL *E. coli* (29.7% (22/74)), (35.1% (26/74)), (52.7% (39/74)), (55.4% (41/74)), (59.5% (44/74)), (64.9% (48/74)), (70.3% (52/74)), (74.3% (55/74)), (54.1% (40/74)) and (56.8% (42/74)) was detected in Jhang, Aseel chickens, Female, birds aged > 12 months, birds reared in large scale farms, free-range husbandry management system, birds feed source (exogenous), farms using disinfected drinking water, birds vaccination in last 6 months and birds in contact with other animals, respectively.

The prevalence of ESBL *E. coli* was significantly associated with the housing system and antibacterial usage in the last 6 months, with *p*-values of 0.004 and 0.005, respectively (Table 3).

Based on the threshold of *p* ≤ 0.25, only six risk factors were included in the binary logistic regression model, including age (*p* = 0.074), housing system (*p* = 0.004), periodic disinfection of drinking water (*p* = 0.07), exposure to other birds and/or animals (*p* = 0.04), extent/level of antimicrobial resistance (AMR) in *E. coli* isolates (*p* = 0.02) and antimicrobial usage of last six months (*p* = 0.005). Binary logistic regression model fitness was tested with an omnibus test (*p* = 0.01) and the Hosmer and Lemeshow test. In the final model, risk factors with *p* ≤ 0.05 were considered significant. The regression model predicted two risk factors to be significantly associated with ESBL *E. coli*; these factors included free-range husbandry management system having *p* = 0.027, OR: 30.00 at 95% CI = 1.471–611.79 and high antimicrobial usage in the last 6 months having *p* = 0.016, OR: 25.175 at 95% CI = 1.817–348.71, as shown in Table 4.

## 3. Discussion

In this study, the overall recovery rate of *E. coli* was found to be 51.3% (164/320). A recent study conducted in Pakistan reported an even higher (82%) recovery rate of *E. coli* in meat and viscera from commercially raised chickens [18]. The estimated mean gathered prevalence of *E. coli* in South-East Asia, including Pakistan, has been reported to be 73% [19]. Multiple factors affect the recovery rate of *E. coli*, including isolation techniques, sample source, host-related factors (chicken breeds, health status and/or vaccination), feed, water, husbandry conditions, ambient temperature, litter management and miscellaneous environmental factors [20].

In the present study, ESBL-producing *E. coli* were detected in 23.1% (74/320) of the samples from backyard poultry. The findings of ESBL *E. coli* prevalence in backyard chickens in this study are not unexpected as they were reported from several countries, i.e., 26.7–38.5% in Nigeria [21], 23.3% in Vietnam [22], 24.9% in Thailand [23] and 13.6% in the USA [15]. ESBL *E. coli* prevalence in Pakistan in commercial chickens were documented to be 38% and 47.6% in samples from the farm environment and chicken meat, respectively [24].

Our findings are consistent with the findings of a previous study conducted in Pakistan that reported 41.1% positive samples from backyard chickens in comparison with 66.9% in commercial broilers [25]. The higher prevalence of MDR and ESBL *E. coli* in commercial chickens compared to the relatively low prevalence in backyard chickens is partly due to the usage pattern of antimicrobials in the two sectors. A recent study reported the high antimicrobial use (AMU) as 96.5 mg/kg of poultry biomass in Pakistan, including the critically important antimicrobial drugs used mainly as growth promoters, prophylaxis and treatment purpose in the commercial chicken sector [26]. However, antibiotics are minimally used as growth promoters in backyard chickens in Pakistan [27]. The high usage of antimicrobial substances in the chicken feed may lead to the selection of resistant strains of bacteria as well as the multidrug resistance phenomenon can emerge as an outcome of co-selection wherein the use of one antibiotic selects the microbes for resistance to another antibiotic compound [28,29].

In the present study, ESBL *E. coli* prevalence was determined in different chicken breeds, including Aseel chickens (8.12%), Golden chickens (5.3%), Naked Neck chickens (5.3%) and Misri chickens (4.4%). The variation of prevalence was insignificant (*p* = 0.96), and no breed predisposition was found. A recent study in Nigeria also showed that the prevalence of ESBL *E. coli* is not dependent on the breeds (broilers and layers) when birds are reared under conditions favoring the growth of resistant pathogens [21].

In the rural areas of Pakistan, backyard chicken farming is practiced as a small, non-intensive type of conventional farming with no or minimal infrastructure and minimal biosecurity and biosafety [30]. Birds of varying ages, breeds and flock sizes (20–50) are reared. Birds remain free-fed in open courtyards in the daytime and are enclosed in small portable wooden or mud enclosures at night. Access to professional veterinary consultancies remains limited. People are often in close contact with these birds, making them vulnerable to contracting zoonotic diseases of public health concern [27].

ESBLs are mostly plasmid-borne and are easily transferred horizontally among bacterial populations. The ESBLs are β-lactamases capable of conferring bacterial resistance to the penicillins (first and second generation), cephalosporins (third generation) and aztreonam by causing structural degradation of these antibiotics; however, these enzymes are inhibited by β-lactamase inhibitors [31]. CTX-M β-lactamases are widely spread among bacteria colonizing multiple species of animals and humans [32]. CTX-M β-lactamases have overtaken the other competing types of β-lactamases, including SHV and TEM, which have been predominant in the recent past [33].

The present study has reported the detection of various ESBL genetic determinants by multiplex PCR detected *bla*_CTX-M_ in 54.05% (40/74), *bla*_TEM_ in 12.2% (9/74) and co-existence of *bla*_CTX-M_ and *bla*_TEM_ genes in 33.8% (25/74) of samples but no *bla*_SHV_. DNA sequence analysis of two *bla*_CTX-M_ gene sequences demonstrated a close sequence similarity with CTX-M-15 type β-lactamase.

Poultry with no clinical symptoms of *E. coli* infection is known to carry CTX-M, while TEM and SHV are often isolated from clinically diseased chickens [9]. As CTX-M-15 has emerged and proven to be of public health significance. It is found in bacteria causing nosocomial infections, animals and the environment, i.e., it is of “One Health” concern [34]. CTX-M-15 does not cause any evident clinical signs in birds [35]. The partial clonal similarity of the CTX-M sequences of poultry origin in the present study (UUJ75596.1 and UTE89519.1) with a CTX-M-15 sequence (AHM26531.1) of a strain of human origin, isolated strain from a tertiary care hospital from Andhra Pradesh, India (https://www.ncbi.nlm.nih.gov/protein/AHM26531.1/ Unpublished data; accessed on 19 May 2023) highlighted the risk of the trans-species spread of these plasmid-borne ESBL genes. As the horizontal transfer of genes is facilitated by wild/migratory birds and animals passing international borders, a global spread of resistant bacteria may be seen in the future.

Apart from treating bacterial infections, antibiotics are used for other purposes in food production, including growth promotion, promotion of feed efficiency and prophylaxis. These practices affect the composition of the intestinal microbiome resulting in the elimination of sensitive strains and the colonization of resistant bacteria [10]. The use of antibiotics in backyard chicken farming is lesser as compared to commercial chicken farming; thus, the presence of antimicrobial-resistant strains as gut colonizers of apparently healthy birds is alarming.

Different AMR profiles were identified for *E. coli* isolates of backyard chickens in this study as 95.1%, 78.6%, 76.8%, 71.3%, 11.6%, 70.1%, 68.9%, 60.4% and 57.3% strains were resistant to commonly used antibiotics of clinical and veterinary significance including tylosin, doxycycline, cefotaxime, enrofloxacin, ciprofloxacin, colistin, trimethoprim/sulfamethoxazole, chloramphenicol and gentamicin, respectively.

A recent study on antimicrobial resistant ESBL *E. coli* isolated from backyard chickens in India found higher resistance levels for co-trimoxazole (91.3%), doxycycline (91.3%), cefotaxime/clavulanic acid (83.8%), norfloxacin (82.7%), colistin (95%) and chloramphenicol (100%). Those isolates were found sensitive to imipenem–EDTA (100.0%), colistin (95%) and gentamicin (43.0%) [36]. The lower resistance to ciprofloxacin (11.6%) compared to enrofloxacin (71.3%) in the present study may be explained because ciprofloxacin is mainly used in humans in Pakistan. The antimicrobial usage (AMU) in the poultry (broiler) industry of Pakistan is higher than 462.57 mg per population correction unit (mg/PCU) and the most commonly used antibiotics during the summer are neomycin (111.39 mg/PCU), doxycycline (91.91 mg/PCU) and tilmicosin (77.22 mg/PCU), while in the winter, doxycycline (196.81 mg/PCU), neomycin (136.74 mg/PCU), and amoxicillin (115.04 mg/PCU) are as the most widely used antibiotics [11,37].

In the present study, the variable multiple antibiotic resistance index (MARI) was estimated for ESBL *E. coli* (0.00–0.84; geometric mean 0.25) and non-ESBL *E. coli* (0.00–0.46; geometric mean 0.17). Multidrug-resistant (MDR) isolates (resistant to ≥3 antibiotics classes) included ESBL *E. coli* (70.27%; MARI 0.23–0.84) and non-ESBL *E. coli* (48.88%; MARI 0.23–0.46). As the present study was conducted in backyard chickens, the findings are comparable to data of 73% MDR ESBL *E. coli* of samples of clinical origin (wounds, stool, phlegm, earwax, blood and lacrimal secretions) [38]. These findings are also similar to a previous report of MARI on the transmission of ESBL *E. coli* from the hospital and municipal sewage to a water basin and to the air at a wastewater treatment plants area and its surroundings in Poland, ranging from 0.45 to 0.63 ESBL *E. coli*, respectively, and a higher MARI of ESBL *E. coli* when compared to non-ESBL *E. coli* [39]. These findings prove the emergence and dissemination potential of *E. coli* strains. The present study predicted two risk factors to be significantly associated with the presence of ESBL *E. coli* in backyard chickens in Pakistan. These factors were the housing system (free-range husbandry management system) (*p* = 0.027, OR: 30.00 at 95% CI = 1.471–611.79) and high antimicrobial usage within the last 6 months (*p* = 0.016, OR: 25.175 at 95% CI = 1.817–348.71). Exposure to other animals or birds (*p* = 0.04) and MDR of isolates (*p* = 0.02) were found to be positively correlated with ESBL *E. coli* in backyard chickens too.

A free-range husbandry management system allows chickens to interact with other animal species. In Pakistan, mostly a mixed type of farming is practiced in rural areas with small units of different animal species, including cattle, buffaloes, sheep, goats and chickens. Chickens often roam around freely and pick up the feed from multiple sources, including kitchen scraps, litter, sewage and animal dung [27,40]. Certain factors, including poor husbandry practices, lack of sanitary conditions, mixed and open farming without application of biosecurity practices, and lack of public awareness, expose these part-time farmers to zoonotic diseases [27].

Easy interspecies transmission of resistant commensal and pathogen bacteria is possible, resulting in gene transfer to host species-adapted strains. A previous study conducted in Vietnam found mixed farming (fish farming and poultry farming) and excessive usage of antimicrobial drugs as the risk factors for the high prevalence of cefotaxime-resistant *E. coli* in chicken farms [41]. Multivariate analysis of risk factors found excessive use of antimicrobial agents exerts selection pressure on *Enterobacteriaceae* to evolve as ESBL producers [42].

## 4. Materials and Methods

### 4.1. Collection and Transport of Cloacal Swab Samples from the Study Area

Specimens for this study were collected from four different backyard chicken breeds indigenous to the Jhang district in the central Punjab province of Pakistan. All four tehsils of the Jhang district, namely, Jhang, Shorkot, Athara Hazari and Ahmad Pur Sial were included. For this cross-sectional study, cloacal swabs (*n =* 320) were collected as eighty samples from each chicken breed, including Aseel, Golden, Misri and Naked Neck chickens. From each tehsil, breed-specific samples (*n* = 20) were included (Table 5).

The sample size was calculated from the formula N = *Z*^2^ *P*(1 − *P*)/*d*^2^ [43]. The estimated prevalence of 51%, 41.1% and 13.7% were considered as reported from previous studies [25,44,45]. Therefore, we considered the mean prevalence (35%) of the previous study. At a 95% confidence level (*Z* = 1.96) and 5% estimated error (*d* = 0.05), the sample size calculated was found to be 349.5. Therefore, in our study, we collected a comparable number of samples *n =* 320 samples (slightly lower than the calculated size considering the logistics).

Backyard farm and household poultry unit owners were pre-consented for participation in this research study. The cloacal swab was collected with a sterile swab stick and placed in a vial containing 1 mL of sterile buffered peptone water (Oxoid, Hampshire, UK). Sample vials were properly labeled and packaged with shipping boxes containing cool gel packs. The specimens were transported to the Microbiology Research Laboratory, Department of Pathobiology, CVAS, Jhang campus, University of Veterinary and Animal Sciences, Lahore, for further investigation.

### 4.2. Isolation and Identification of ESBL Producing E. coli

The samples were enriched by inoculating 500 µL of the buffered peptone water containing the cloacal swab in 10 mL of Luria Bertani (LB) broth (Invitrogen, Fisherscientific, Leicestershire, UK) supplemented with cefotaxime (4 mg/L) (Oxoid, UK) and incubated at 37 °C for 24 h. A loopful of LB broth was streaked directly onto MacConkey agar (Oxoid, Hampshire, UK) and supplemented with 4 mg/L cefotaxime as previously described [46]. Based on colony morphology, typical discrete colonies were further subcultured in nutrient broth to obtain a pure culture. Pure cultures were confirmed as *E. coli* using analytical profile index (API)-20E (bioMérieux, Craponne, France) test strips as per the manufacturer’s instructions. The isolates were confirmed as Extended Spectrum β-lactamase (ESBL) producers via Double Disc Synergy Test (DDST) according to CLSI 2020 [47]. DDST was performed by swabbing a loopful of broth of confirmed *E. coli* cultures that were eight hours incubated onto Mueller–Hinton agar (MHA) (Oxoid, Hampshire, UK) plates. Cefotaxime 30 μg (CTX-30) and Amoxicillin/clavulanic acid 30 μg (AMC-30) discs (Oxoid, Hampshire, UK) were placed at a 20 mm center-to-center distance to center onto the MHA plates and incubated at 37 °C for 24 h. *E. coli* (ATCC BAA-2326) was used as ESBL control. The expansion of the zone of inhibition of CTX-30 towards the AMC-30 disc was considered a positive DDST and confirmed as ESBL *E. coli*.

### 4.3. Antimicrobial Susceptibility and Multiple Antibiotic Resistance Index (MARI) Profiling of E. coli

The antimicrobial susceptibility test (AST) was performed for confirmed *E. coli* as described by CLSI M100s of Clinical Laboratory Standards Institute 2020 [47]. Standardized suspensions of pure broth cultures equivalent in turbidity to 0.5 McFarland standard (Becton Dickinson, New Jersey, USA) were swabbed onto Mueller–Hinton agar (Oxoid, Hampshire, UK) plates. Thirteen antimicrobial agents were used including penicillin (amoxicillin/clavulanic acid, 30 μg), cephalosporin (cefotaxime, 30 µg), polymyxin (colistin, 10 µg), phenicol (chloramphenicol. 30 µg), a quinolone (enrofloxacin, 5 μg, ciprofloxacin, 5 μg and norfloxacin 5 μg), aminoglycosides (gentamicin, 10 μg, neomycin, 30 µg), a macrolide (tylosin 30 µg), sulfonamide (trimethoprim/sulfamethoxazole 1.25/23.75 μg), tetracycline (doxycycline, 30 µg) and carbapenem (imipenem, 10 μg). Following the incubation at 37 °C for 24 h, the diameter of the zone of inhibition was recorded in millimeters (mm) and interpreted as resistant, intermediate or sensitive according to CLSI 2020 [47]. *E. coli* (ATCC 8739) was used for the antibiotic sensitivity test (AST) control strain.

The multiple antibiotic resistance index (MARI) was determined for resistant *E. coli* isolates as a ratio of the number of antibiotics to which an isolate was found resistant and the total number of antibiotics used (*n =* 13) [48]. The MARI was used as an indicator of the extent of multidrug resistance among both ESBL and non-ESBL *E. coli* isolates. Most of the selected antibiotics are used in veterinary prescriptions and food animal production, including the poultry sector. However, some of the selected antibiotics are exclusively used in human medicine (e.g., imipenem and cefotaxime). The panel of antibiotics was finally selected under the one-health approach to represent antibiotics of both human and veterinary importance [37,49].

### 4.4. Genomic DNA Extraction and Purification

Deoxyribose nucleic acid (DNA) was extracted from the overnight incubated nutrient broth samples of *E. coli* cultures. PureLink™ Genomic DNA Mini Kit (Thermo Fisher Scientific, Waltham, MA, USA) was used for DNA extraction as per the manufacturer’s instructions. Briefly, broth culture (1 mL) was centrifuged at 14,000 rpm for 10 min using a refrigerated centrifuge (Hermle, Gosheim, Germany). The pellet was incubated with a digestion solution and proteinase K. RNase A solution was used to degrade RNA contamination. Lysis solution (200 μL) was added and the mixture was homogenized by using a vortex mixer (Witeg, Wertheim, Germany) followed by the addition of ice-cold 50% ethanol and re-homogenization. The lysate was transferred to a spin column and treated with wash buffer I and centrifuged. Wash buffer II was added and centrifugation followed. Elution buffer (80 μL) was added to the spin column, incubated for two minutes at room temperature and centrifuged at 14,000 rpm for 6 min to collect the final purified DNA. The DNA was stored at −20 °C until used.

### 4.5. Detection of Associated ESBL Genes Using Multiplex PCR

Three different types of plasmid-borne acquired extended-spectrum β-lactamases (ESBL), including Cefotaxime-hydrolysing β-lactamase-Munich (CTX-M), Sulfhydryl reagent variable (SHV) and Temoneira β-lactamase (TEM) were detected by multiplex polymerase chain reaction (mPCR) targeting *bla*_CTX-M_, *bla*_SHV_ and *bla*_TEM_ genes [50]. The primer-pair sequences (bla-SHV.SE/AS; TEM-164.SE and TEM-165.AS; universal CTX-M-U1/U2) used in the multiplex PCR assay, primer sequences and expected PCR amplicon sizes are given in Table 6.

Multiplex PCR reaction mixture (50 µL) was prepared by using 25 µL master mix Dream Taq Green 2x, (Thermo Fisher Scientific, USA), 4 µL template DNA, 2 µL each primer (10 picomole/µL) and made up to 50 µL by adding nuclease-free water. Amplification was carried out in a thermal cycler (Biorad, Hercules, CA, USA), and PCR amplification conditions were as follows: initial denaturation at 95 °C for 15 min followed by 30 cycles of denaturation at 94 °C for 30 s, annealing at 60 °C for 30 s, extension at 72 °C for 2 min and a single final extension step at 72 °C for 10 min. Positive control DNA templates for PCR were maintained in-house at our laboratory and were verified through sequencing and BLAST functionality of NCBI. PCR products were analyzed in 1.2% agarose gels in tris borate EDTA (TBE) buffer. The gel was run at 100 V for 40 min and stained with ethidium bromide (0.5 μg/mL). Gel images were obtained with a gel documentation system (Syngene, Cambridge, UK) by using Genesys software.

### 4.6. DNA Sequencing of bla_CTX-M_ Gene Amplicon

Amplicons of the *bla*_CTX-M_ gene were sequenced by the Sanger dideoxy method. Briefly, the *bla*_CTX-M_ gene amplicons of all the isolates with a length of approximately 593 bp were excised from the gel, and DNA was purified using the QIAamp Gel Extraction Kit (Qiagen, Hilden, Germany) according to the manufacturer’s recommendations. Cycle sequencing was done with different sequencing primers (Table 6) using BigDye Terminator v1.1 Cycle Sequencing Kit (Applied Biosystems, Darmstadt, Germany) according to the recommendations of the manufacturer. Sequencing products were analyzed with a Genetic Analyzer ABI PRISM 3130 (Applied Biosystems). The *bla*_CTX-M_ gene sequences were analyzed to identify the most parsimonious relationships. A phylogenetic tree was constructed with the maximum likelihood method with bootstraps (1000) and the Jones–Taylor–Thornton (JTT) model by using MEGA 11 software (64-bit) [51].

The cefotaxime-hydrolyzing β-lactamase amino acid sequence data for corresponding nucleotide data are also accessible at the NCBI web portal with accession numbers UTE89519.1 and UUJ75596.1, respectively. The amino acid sequences (UTE89519.1, UUJ75596.1) were analyzed via the BLAST-p online tool of NCBI and a phylogenetic analysis of sequences was made by including more similar β-lactamase sequences CTX-M-14 (BAI68282.1), CTX-M-9 (AAZ30046.1), CTX-M-2 (AMQ12632.1, AMQ12636.1), CTX-M-15 (AHM26531.1), less similar sequences SHV (AAV91761.1, AAV91760.1), TEM (AMO65331.1, AMO65330.1) and outgroup sequences OXA-1 (AGS09440.1, AGS09441.1).

### 4.7. Statistical Analysis of Associated Risk Factors

A semi-structured survey was designed for data collection related to the backyard chicken units in line with prevailing husbandry conditions. The potential risk factors, including location, chicken breed, sex, age, size of the farm, housing system, feeding resources, disinfection of drinking water, history of vaccination, contact with other animals and antimicrobial usage in the last 6 months, were statistically analyzed. Data were analyzed for an assessment of the risk factors related to ESBL *E. coli*. Chi-square (X^2^) and Fisher exact tests were performed to determine the association between the outcome variable (ESBL *E. coli*) and twelve predictor variables (risk factors) in univariable analysis. Predictor variables with *p* ≤ 0.25 were selected for further analysis by using a binary logistic regression model. In the logistic regression model, the dichotomous dependent variable was coded as “0” for non-ESBL *E. coli* and “1” for ESBL *E. coli* to predict the significant influence of pre-selected risk factors [12]. All statistical analyses were made by using the software IBM SPSS Statistics version 25.

## 5. Conclusions

This study provides information about the prevalence and genetic characterization of ESBL *E. coli* in backyard chickens of the Jhang district, Punjab province, Pakistan. Backyard chicken units are potential reservoirs for multidrug-resistant bacteria posing a severe threat to the community, environment and food chain. To the best of the knowledge of the authors, the present study is the first one to characterize ESBL genes in the *E. coli* population and to analyze risk factors in backyard chickens in Pakistan.

Close contact with backyard chickens has to be considered a risk for contracting resistant strains of *E. coli*. Two mobile genetic elements are responsible for the production of β lactamases (*bla*_CTX-M_ and *bla*_TEM_) were detected, while *bla*_SHV_ was not detected. The nucleotide sequence analysis confirmed the sequence homology of β-lactamases of backyard chicken origin with the β-lactamases of clinical origin. ESBL production has been correlated with drug usage. The use of antimicrobial agents for growth promotion, prophylaxis and treatment, as well as free-range husbandry practices encouraging frequent chicken interactions with other species of farm animals, have been found as potential risk factors. Conclusively, backyard chicken flocks can serve as a potential reservoir of ESBL *E. coli*. This study advocates for strengthening bio-surveillance systems for backyard chickens to limit the emergence of antimicrobial-resistant *Enterobacteriaceae*. These data are also crucial for making informed decisions related to food safety, food security and general public health.

Considering the findings of the present study, it was recommended for policymakers effectively control the emergence of antimicrobial resistance in the backyard chicken sector in Pakistan. These recommendations include but are not limited to designing and implementing the antimicrobial drugs usage policy for backyard poultry farmers to discourage the injudicious use of antimicrobial substances. Use of antimicrobials as growth promoters must be banned by creating awareness and providing excess alternatives such as probiotics, prebiotics, essential oils, organic acids and phytobiotics. Moreover, there is a dire need to improve husbandry practices, chicken housing management and implementation of strict bio-risk management systems for backyard chicken farming in Pakistan.

The present study was limited to just one district of the Punjab province in Pakistan. Further research is needed to cover a large sampling area for backyard chicken units. In Pakistan, backyard poultry farming is emerging and supported by the federal government via the project of the prime minister’s initiative for backyard poultry. However, whole genome sequencing-based characterization of genetic determinants for antimicrobial resistance is needed to tailor the best bio-surveillance and initiatives taken under the One-Health approach can be of significance for monitoring and controlling antimicrobial resistance in backyard chickens.

Further research is warranted to discover new alternatives of antibiotics and to evaluate the efficiency of existing antibiotic alternative substances as replacers of antibiotics in an effort to reduce the pace of emerging antimicrobial resistance.

## Figures and Tables

**Table 1:** Antibacterial sensitivity profile of *E. coli* isolates (*n* = 164) recovered from backyard chickens, Jhang, Pakistan.

Drug Class	Antibiotic Agent	No. of Isolates		
		Resistant *n* (%)	Intermediate *n* (%)	Susceptible *n* (%)
Penicillin	Amoxicillin/Clavulanate	58 (35.4%)	7 (4.3%)	99 (60.4%)
Cephalosporin	Cefotaxime (3rd Generation)	126 (76.8%)	4 (2.4%)	34 (20.7%)
Phenicol	Chloramphenicol	99 (60.4%)	9 (5.5%)	56 (34.1%)
Polymyxin	Colistin	115 (70.1%)	11 (6.7%)	38 (23.2%)
Quinolone/Fluoroquinolone	Enrofloxacin	117 (71.3%)	13 (7.9%)	34 (20.7%)
	Ciprofloxacin	19 (11.6%)	8 (4.9%)	137 (84%)
	Norfloxacin	71 (43.3%)	6 (3.7%)	87 (53.1%)
Aminoglycosides	Gentamicin	94 (57.3%)	8 (4.9%)	62 (37.8%)
	Neomycin	20 (12.2%)	7 (4.3%)	137 (83.5%)
Carbapenem	Imipenem	16 (9.8%)	10 (6.1%)	138 (84.1%)
Tetracycline	Doxycycline	129 (78.6%)	17 (10.4%)	18 (10.9%)
Sulfonamide	Trimethoprim/sulfamethoxazole	113 (68.9%)	21 (12.8%)	30 (18.3%)
Macrolide	Tylosin	156 (95.1%)	3 (1.8%)	5 (3.0%)

**Table 2:** Multiple antibiotic resistance index (MARI) of ESBL and non-ESBL *E. coli* strains.

ESBL *E. coli* (*n =* 74)	MAR Index *	Non-ESBL *E. coli* (*n =* 90)	MAR Index
3 (4.05%)	0.84	9 (10%)	0.46
1 (1.35%)	0.61	11 (12.2%)	0.38
6 (8.11%)	0.46	21 (23.3%)	0.23
16 (21.62%)	0.38	18 (20%)	0.15
26 (35.13%)	0.23	31 (34.4%)	0
9 (12.16%)	0.07	-	-
13 (17.57%)	0	-	-
Geometric mean	0.25	Geometric mean	0.17

* Multiple antibiotic resistance index (MARI) was determined for resistant *E. coli* isolates as a ratio of the number of antibiotics to which an isolate was found resistant and the total number of antibiotics used (*n* = 13).

**Table 3:** Potential risk factors associated with the presence of ESBL *E. coli* in backyard chickens evaluated by using the chi-square test.

Predictor Variables as Risk Factors		Total *E. coli*	Categorical Response Variable		Chi-Square (X^2^) Statistics	*p*-Value
			ESBL *E. coli* (Code: 1)	Non-ESBL *E. coli* (Code: 0)		
		(*n* = 164)	(*n =* 74)	(*n =* 90)		
Location	Ahmad Pur Sial	46 (28%)	16 (22.2%)	30 (33.3%)	2.77	0.428
	Shorkot	36 (21.9%)	18 (24.3%)	18 (20%)		
	Athara Hazari	37 (22.5%)	18 (24.3%)	19 (21.1%)		
	Jhang	45 (27.4%)	22 (29.7%)	23 (25.5%)		
Chicken Breed	Aseel chicken	50 (30.5%)	26 (35.1%)	24 (26.6%)	1.597	0.66
	Golden chicken	43 (26.2%)	17 (23%)	26 (28.9%)		
	Misri chicken	33 (20.1%)	14 (18.9%)	19 (21.1%)		
	Naked neck chicken	38 (23.2%)	17 (23%)	21 (23.3%)		
Sex	Male	84 (51.2%)	35 (47.3%)	49 (54.4%)	0.830	0.362
	Female	80 (48.8%)	39 (52.7%)	41 (45.5%)		
Age	<6 months	40 (24.4%)	14 (18.9%)	26 (28.9%)	5.21	0.074
	≥6 months–12 months	49 (29.9%)	19 (25.7%)	30 (33.3%)		
	> 12 months	75 (45.7%)	41 (55.4%)	34 (37.8%)		
Size of farm/unit birds	Small (<50 birds)	61 (37.2%)	30 (40.5%)	31 (34.4%)	0.646	0.422
	Large (≥50 birds)	103 (62.8%)	44 (59.5%)	59 (65.5%)		
Housing system	Strict Cage system	78 (47.5%)	26 (35.1%)	52 (57.8%)	8.348	0.004
	Free-range husbandry management system	86 (52.4%)	48 (64.9%)	38 (42.2%)		
Feeding resource	Commercial feed	52 (31.7%)	22 (29.7%)	30 (33.3%)	0.244	0.622
	Exogenous (Free picking)	112 (68.3%)	52 (70.3%)	60 (66.7%)		
Disinfection of drinking water	Yes	132 (80.5%)	55 (74.3%)	77 (85.5%)	3.262	0.07
	No	32 (19.5%)	19 (25.7%)	13 (14.4%)		
Vaccination in last 6 months	Yes	88 (53.6%)	40 (54.1%)	48 (53.3%)	0.008	0.927
	No	76 (46.3%)	34 (46%)	42 (46.7%)		
Contact with animals/birds	No	85 (51.8%)	32 (32.2%)	53 (58.9%)	3.98	0.04
	Yes	79 (48.2%)	42 (56.8%)	37 (41.1%)		
Antimicrobial Resistance Level	Non-MDR	72 (43.9%)	25 (33.8%)	47 (52.2%)	5.60	0.02
	MDR	92 (56.1%)	49 (66.2%)	43 (47.8%)		
Antibacterial usage in last 6 months	Low (≤5 mg/kg/week)	49 (29.9%)	15 (20.3%)	34 (37.8%)	10.53	0.005
	Moderate (>5–10 mg/kg/week)	54 (32.9%)	22 (30%)	32 (35.5%)		
	High (>10 mg/kg/week)	61 (37.2%)	37 (50%)	24 (26.7%)		

**Table 4:** Potential risk factors associated with ESBL *E. coli* in backyard chickens evaluated by using binary logistic regression analysis.

Pre-Selected Risk Factors (Codes ^1^)		Categorical Response Variable		β Coefficient	SE ^2^	Prevalence Odd Ratio (OR)	(95% Confidence Level Range)	*p*-Value
		ESBL *E. coli*	Non-ESBL *E. coli*					
Age	<6 m. (0)	14	26	Baseline				0.418
	≥6 m.–12 m. (1)	19	30	−1.460	1.111	0.232	0.026–2.050	0.189
	>12 m. (2)	41	34	−1.278	1.708	0.279	0.010–7.926	0.454
Housing system	Strict Cage system (0)	26	52	Baseline				
	Free-range husbandry management system (1)	48	38	3.401	1.538	30.00	1.471–611.79	0.027
Disinfection of drinking water	Yes (0)	55	77	Baseline				
	No (1)	19	13	−0.113	0.525	0.893	0.319–2.50	0.830
Exposure to other animals/birds	No (0)	32	53	Baseline				
	Yes (1)	42	37	−2.890	1.581	0.056	0.003–1.232	0.068
Antimicrobial Resistance Level	Non-MDR (0)	25	47	Baseline				
	MDR (1)	49	43	−1.347	1.173	0.260	0.026–2.593	0.251
Antibacterial usage in last 6 months	Low (0) (≤5 mg/kg/week)	15	34	Baseline				0.038
	Moderate (1) (>5–10 mg/kg/week)	22	32	1.817	1.141	6.154	0.658–57.59	0.111
	High (2) (>10 mg/kg/week)	37	24	3.226	1.341	25.175	1.817–348.71	0.016
Constant				−0.619	0.331	0.538		0.062

^1^ Codes: (0, 1, 2); ^2^ Standard Error.

**Table 5:** Summary of cloacal swabs collected from different backyard chicken breeds in the Jhang district, Punjab, Pakistan.

Tehsil	No. of Cloacal Swabs				
	Aseel Chicken	Golden Chicken	Misri Chicken	Naked Neck Chicken	Total
Jhang	20	20	20	20	80
Shorkot	20	20	20	20	80
Athara Hazari	20	20	20	20	80
Ahmad Pur Sial	20	20	20	20	80
Total	80	80	80	80	320

**Table 6:** Primers used for the detection of ESBL genes.

Target Gene	Primer Name	Primer Sequences (5′ to 3′)	Product Size
*bla*_TEM_	TEM-SE	TCGCCGCATACACTATTCTCAGAATGA	445 bp
	TEM-AS	ACGCTCACCGGCTCCAGATTTAT	
*bla*_CTX-M_	CTX-M-U1	ATGTGCAGYACCAGTAARGTKATGGC	593 bp
	CTX-M-U2	TGGGTRAARTARGTSACCAGAAYCAGCGG	
*bla*_SHV_	SHV-SE	ATGCGTTATATTCGCCTGTG	747 bp
	SHV-AS	TGCTTTGTTATTCGGGCCAA	

## Data Availability

The data presented in this study are available on request from the corresponding author.

## References

[1] Rahman S., Kesselheim A.S., Hollis A. (2023). Persistence of resistance: A panel data analysis of the effect of antibiotic usage on the prevalence of resistance. J. Antibiot..

[2] Murray C.J.L., Ikuta K.S., Sharara F., Swetschinski L., Aguilar G.R., Gray A., Han C., Bisignano C., Rao P., Wool E. (2022). Global burden of bacterial antimicrobial resistance in 2019: A systematic analysis. Lancet.

[3] Sarowska J., Futoma-Koloch B., Jama-Kmiecik A., Frej-Madrzak M., Ksiazczyk M., Bugla-Ploskonska G., Choroszy-Krol I. (2019). Virulence factors, prevalence and potential transmission of extraintestinal pathogenic *Escherichia coli* isolated from different sources: Recent reports. Gut Pathog..

[4] Kim Y.B., Yoon M.Y., Ha J.S., Seo K.W., Noh E.B., Son S.H., Lee Y.J. (2020). Molecular characterization of avian pathogenic *Escherichia coli* from broiler chickens with colibacillosis. Poult. Sci..

[5] Silva N., Costa L., Gonçalves A., Sousa M., Radhouani H., Brito F., Igrejas G., Poeta P. (2012). Genetic characterisation of extended-spectrum β-lactamases in *Escherichia coli* isolated from retail chicken products including CTX-M-9 containing isolates: A food safety risk factor. Br. Poult. Sci..

[6] Castanheira M., Simner P.J., Bradford P.A. (2021). Extended-spectrum β-lactamases: An update on their characteristics, epidemiology and detection. JAC Antimicrob. Resist..

[7] Tooke C.L., Hinchliffe P., Bragginton E.C., Colenso C.K., Hirvonen V.H.A., Takebayashi Y., Spencer J. (2019). beta-Lactamases and beta-Lactamase Inhibitors in the 21st Century. J. Mol. Biol..

[8] Founou L.L., Founou R.C., Essack S.Y. (2016). Antibiotic resistance in the food chain: A developing country-perspective. Front. Microbiol..

[9] Olsen R.H., Bisgaard M., Lohren U., Robineau B., Christensen H. (2014). Extended-spectrum beta-lactamase-producing *Escherichia coli* isolated from poultry: A review of current problems, illustrated with some laboratory findings. Avian Pathol..

[10] Castanon J.I.R. (2007). History of the use of antibiotic as growth promoters in European poultry feeds. Poult. Sci..

[11] Umair M., Tahir M.F., Ullah R.W., Ali J., Siddique N., Rasheed A., Akram M., Zaheer M.U., Mohsin M. (2021). Quantification and trends of antimicrobial use in commercial broiler chicken production in Pakistan. Antibiotics.

[12] Ibrahim R.A., Cryer T.L., Lafi S.Q., Abu Basha E., Good L., Tarazi Y.H. (2019). Identification of *Escherichia coli* from broiler chickens in Jordan, their antimicrobial resistance, gene characterization and the associated risk factors. BMC Vet. Res..

[13] CDC Keeping Backyard Chickens and Other Poultry. https://www.cdc.gov/healthypets/pets/farm-animals/backyard-poultry.html.

[14] Pohjola L., Nykasenoja S., Kivisto R., Soveri T., Huovilainen A., Hanninen M.L., Fredriksson-Ahomaa M. (2016). Zoonotic Public Health Hazards in Backyard Chickens. Zoonoses Public Health.

[15] Shah D.H., Board M.M., Crespo R., Guard J., Paul N.C., Faux C. (2020). The occurrence of *Salmonella*, extended-spectrum beta-lactamase producing *Escherichia coli* and carbapenem resistant non-fermenting Gram-negative bacteria in a backyard poultry flock environment. Zoonoses Public Health.

[16] Sharma B. (2010). Poultry production, management and bio-security measures. J. Agri. Environ..

[17] GOP, Economic Adviser’s Wing (2022). Agriculture. Pakistan Economic Survey 2021–22 Chapter 2.

[18] Liaqat Z., Khan I., Azam S., Anwar Y., Althubaiti E.H., Maroof L. (2022). Isolation and molecular characterization of extended spectrum beta lactamase producing *Escherichia coli* from chicken meat in Pakistan. PLoS ONE.

[19] Dawadi P., Bista S., Bista S. (2021). Prevalence of colistin-resistant *Escherichia coli* from poultry in south asian developing countries. Vet. Med. Int..

[20] Dandachi I., Sokhn E.S., Dahdouh E.A., Azar E., El-Bazzal B., Rolain J.M., Daoud Z. (2018). Prevalence and characterization of multi-drug-resistant gram-negative bacilli isolated from lebanese poultry: A nationwide study. Front. Microbiol..

[21] Kwoji I.D., Musa J.A., Daniel N., Mohzo D.L., Bitrus A.A., Ojo A.A., Ezema K.U. (2019). Extended-spectrum beta-lactamase-producing *Escherichia coli* in chickens from small-scale (backyard) poultry farms in Maiduguri, Nigeria. Int. J. One Health.

[22] Nakayama T., Jinnai M., Kawahara R., Diep K.T., Thang N.N., Hoa T.T., Hanh L.K., Khai P.N., Sumimura Y., Yamamoto Y. (2017). Frequent use of colistin-based drug treatment to eliminate extended-spectrum beta-lactamase-producing *Escherichia coli* in backyard chicken farms in Thai Binh Province, Vietnam. Trop. Anim. Health Prod..

[23] Aworh M.K., Kwaga J., Okolocha E., Harden L., Hull D., Hendriksen R.S., Thakur S. (2020). Extended-spectrum β-lactamase-producing *Escherichia coli* among humans, chickens and poultry environments in Abuja, Nigeria. One Health Outlook..

[24] Rahman S. (2019). Incidence of ESBL-producing-*Escherichia coli* in poultry farm environment and retail poultry meat. Pak. Vet J..

[25] Kamboh A.A., Shoaib M., Abro S.H., Khan M.A., Malhi K.K., Yu S.Q. (2018). Antimicrobial resistance in *Enterobacteriaceae* isolated from liver of commercial broilers and backyard chickens. J. Appl. Poult. Res..

[26] Umair M., Orubu S., Zaman M.H., Wirtz V.J., Mohsin M. (2022). Veterinary consumption of highest priority critically important antimicrobials and various growth promoters based on import data in Pakistan. PLoS ONE.

[27] Ahmed T., Ameer H.A., Javed S. (2021). Pakistan’s backyard poultry farming initiative: Impact analysis from a public health perspective. Trop. Anim. Health Prod..

[28] Diarra M.S., Malouin F. (2014). Antibiotics in Canadian poultry productions and anticipated alternatives. Front. Microbiol..

[29] Wales A.D., Davies R.H. (2015). Co-selection of resistance to antibiotics, biocides and heavy metals, and Its relevance to foodborne pathogens. Antibiotics.

[30] Pym R. (2010). Poultry Housing and Management in Developing Countries, Management and Housing of Semi-Scavenging Flocks.

[31] Paterson D.L., Bonomo R.A. (2005). Extended-spectrum beta-lactamases: A clinical update. Clin. Microbiol. Rev..

[32] Bevan E.R., Jones A.M., Hawkey P.M. (2017). Global epidemiology of CTX-M β-lactamases: Temporal and geographical shifts in genotype. J. Antimicrob. Chemother..

[33] Ramos S., Silva V., Dapkevicius M.L.E., Canica M., Tejedor-Junco M.T., Igrejas G., Poeta P. (2020). *Escherichia coli* as commensal and pathogenic bacteria among food-producing animals: Health implications of Extended Spectrum beta-lactamase (ESBL) production. Animals.

[34] Wyres K.L., Hawkey J., Hetland M.A.K., Fostervold A., Wick R.R., Judd L.M., Hamidian M., Howden B.P., Löhr I.H., Holt K.E. (2019). Emergence and rapid global dissemination of CTX-M-15-associated *Klebsiella pneumoniae* strain ST307. J. Antimicrob. Chemother..

[35] Valentin L., Sharp H., Hille K., Seibt U., Fischer J., Pfeifer Y., Michael G.B., Nickel S., Schmiedel J., Falgenhauer L. (2014). Subgrouping of ESBL-producing *Escherichia coli* from animal and human sources: An approach to quantify the distribution of ESBL types between different reservoirs. Int. J. Med. Microbiol..

[36] Chowdhury M., Bardhan R., Pal S., Banerjee A., Batabyal K., Joardar S.N., Mandal G.P., Bandyopadhyay S., Dutta T.K., Sar T.K. (2022). Comparative occurrence of ESBL/AmpC beta-lactamase-producing *Escherichia coli* and *Salmonella* in contract farm and backyard broilers. Lett. Appl. Microbiol..

[37] Mohsin M., Van Boeckel T.P., Saleemi M.K., Umair M., Naseem M.N., He C., Khan A., Laxminarayan R. (2019). Excessive use of medically important antimicrobials in food animals in Pakistan: A five-year surveillance survey. Glob. Health Action.

[38] Masoud S.M., Abd El-Baky R.M., Aly S.A., Ibrahem R.A. (2021). Co-existence of certain ESBLs, MBLs and plasmid mediated quinolone resistance genes among MDR *E. coli* isolated from different clinical specimens in Egypt. Antibiotics.

[39] Korzeniewska E., Korzeniewska A., Harnisz M. (2013). Antibiotic resistant *Escherichia coli* in hospital and municipal sewage and their emission to the environment. Ecotox. Environ. Saf..

[40] Sahota A.W., Bhatti B.M. (2003). Productive performance of Desi field chickens as affected under deep litter system. Pak. J. Vet Res..

[41] Nguyen V.T., Carrique-Mas J.J., Ngo T.H., Ho H.M., Ha T.T., Campbell J.I., Nguyen T.N., Hoang N.N., Pham V.M., Wagenaar J.A. (2015). Prevalence and risk factors for carriage of antimicrobial-resistant *Escherichia coli* on household and small-scale chicken farms in the Mekong Delta of Vietnam. J. Antimicrob. Chemother..

[42] Reuland E.A., Al Naiemi N., Kaiser A.M., Heck M., Kluytmans J.A., Savelkoul P.H., Elders P.J., Vandenbroucke-Grauls C.M. (2016). Prevalence and risk factors for carriage of ESBL-producing *Enterobacteriaceae* in Amsterdam. J. Antimicrob. Chemother..

[43] Daniel W.W. (1999). Biostatistics: A Foundation for Analysis in the Health Sciences.

[44] Shoaib M., Kamboh A.A., Sajid A., Mughal G.A., Leghari R.A., Malhi K.K., Bughio S., Ali A., Alam S., Khan S. (2016). Prevalence of extended spectrum beta-lactamase producing enterobacteriaceae in commercial broilers and backyard chickens. Adv. Anim. Vet. Sci..

[45] Umair M., Mohsin M., Ali Q., Qamar M.U., Raza S., Ali A., Guenther S., Schierack P. (2019). Prevalence and genetic relatedness of extended spectrum-β-lactamase-producing *Escherichia coli* among humans, cattle, and poultry in Pakistan. Microb. Drug Resist..

[46] Eshrati B., Baradaran H.R., Motevalian S.A., Majidpour A., Boustanshenas M., Soleymanzadeh Moghadam S., Moradi Y. (2020). Investigating the relationship between extended spectrum β-lactamase producing *Escherichia coli* in the environment and food chains with the presence of this infection in people suspected of septicemia: Using the fuzzy set qualitative comparative analysis. J. Environ. Health Sci. Eng..

[47] CLSI (2020). Performance Standards for Antimicrobial Susceptibility Testing. CLSI Supplement M100s.

[48] Davis R., Brown P.D. (2016). Multiple antibiotic resistance index, fitness and virulence potential in respiratory *Pseudomonas aeruginosa* from Jamaica. J. Med. Microbiol..

[49] MNHS (2017). Antimicrobial Resistance National Action Plan Pakistan.

[50] Monstein H.J., Ostholm-Balkhed A., Nilsson M.V., Nilsson M., Dornbusch K., Nilsson L.E. (2007). Multiplex PCR amplification assay for the detection of *bla*_SHV_, *bla*_TEM_ and *bla*_CTX-M_ genes in *Enterobacteriaceae*. Apmis.

[51] Wollenberg K.R., Atchley W.R. (2000). Separation of phylogenetic and functional associations in biological sequences by using the parametric bootstrap. Proc. Natl. Acad. Sci. USA.

